# Predicting treatment response of patients with extranodal natural killer/T‐cell lymphoma based on levels of PD‐L1 mRNA and soluble PD‐L1

**DOI:** 10.1002/hon.2758

**Published:** 2020-07-09

**Authors:** Yu Feng, Caixia Jing, Xinmei Yu, Xia Cao, Caigang Xu

**Affiliations:** ^1^ Department of Hematology/Hematology Research Laboratory, West China Hospital Sichuan University Chengdu China

**Keywords:** disease staging, extranodal natural killer/T‐cell lymphoma, programmed cell death ligand 1, programmed cell death receptor 1, treatment response assessment

## Abstract

Appropriate biomarkers may help predict patient response to treatment for extranodal natural killer/T‐cell lymphoma (ENKTL), a subtype of non‐Hodgkin's lymphoma in China. Programmed cell death receptor 1 (PD‐1) and its ligand (PD‐L1) have been investigated in various tumors. However, few studies have addressed expression of PD‐1/PD‐L1 in peripheral blood of ENKTL patients. To identify novel peripheral blood biomarkers for diagnosis and treatment of ENKTL, we retrospectively examined 89 healthy volunteers, 49 patients with ENKTL and 74 patients with diffuse large B‐cell lymphoma treated at West China Hospital from September 2017 to September 2018. Both patient groups showed significantly higher expression of PD‐1 and PD‐L1 on CD4+ T cells, higher levels of PD‐L1 mRNA in peripheral blood mononuclear cells (PBMCs) and higher levels of soluble PD‐L1 in plasma than healthy volunteers (*P* < .05). In ENKTL patients, levels of PD‐L1 mRNA and soluble PD‐L1 were related to disease stage, level of lactate dehydrogenase, lymphocyte count, and copies of Epstein‐Barr genome in blood. Levels of PD‐L1 mRNA and soluble PD‐L1 were similar between healthy volunteers and ENKTL patients who showed complete remission after treatment, and uni‐ and multivariate analyses identified soluble PD‐L1 as a predictor of treatment response in ENKTL patients. Our results suggest that the levels of PD‐L1 mRNA in PBMCs and soluble PD‐L1 in plasma are useful for ENKTL staging and prediction of treatment response.

## 1 INTRODUCTION

Extranodal natural killer/T‐cell lymphoma (ENKTL) is characterized by high invasion, rapid progress, and poor prognosis.^1^ Lymphoma cells interact with bystander cells in tumor tissue to form a microenvironment that maintains cancer cell survival and promotes their proliferation through evasion of immune responses. The signaling pathway involving programmed cell death receptor 1(PD‐1) and its ligand PD‐L1 may be one mechanism of immune evasion.^2^

PD‐1 protein is an immune‐checkpoint receptor expressed on activated T and B lymphocytes, to which PD‐L1 on the surface of tumor cells can bind, which inhibits lymphocyte proliferation and activation.^3^ This phenomenon has been demonstrated in melanoma,^2^ gastric cancer,^4^ esophageal cancer,^5^ pancreatic cancer,^6^ liver cancer,^7^ kidney cancer,^8^ and ovarian cancer.^9^ In fact, the PD‐1/PD‐L1 signaling pathway helps regulate the peripheral blood immune response in non‐small cell lung cancer,^10^ oral squamous cell carcinoma,^11^ gastric cancer,^12^ and ovarian cancer.^13^ PD‐1 level on CD4+ or CD8+T cells in peripheral blood is elevated in patients with Hodgkin's lymphoma^14^ and chronic lymphocytic leukemia.^15^

Whether PD‐1/PD‐L1 signaling is involved in ENKTL is unclear. Two studies have reported that elevated levels of soluble PD‐L1 (sPD‐L1) in serum correlate with poor prognosis in ENKTL.^16^, ^17^

Therefore, we examined the expression of PD‐1/PD‐L1 on circulating lymphocytes as well as levels of PD‐L1 mRNA in peripheral blood mononuclear cells (PBMCs) and sPD‐L1 in plasma in ENKTL patients. Our results show that the levels of PD‐L1 mRNA in PBMCs and sPD‐L1 in plasma are useful for ENKTL staging and prediction of treatment response. The same results were not obtained for patients with another type of lymphoma, diffuse large B‐cell lymphoma (DLBCL), suggesting that the two biomarkers may show some specificity for ENKTL.

## 2 MATERIALS AND METHODS

### 2.1 Study population and treatment

This retrospective study involved 49 patients with ENKTL and 74 with DLBCL who were initially treated at West China Hospital from September 2017 to September 2018. Diagnosis was made according to the 2008 WHO Classification of Hematopoietic and Lymphoid Tissue Tumors.^18^ Patients were included if they had not received any treatment prior to initial diagnosis. Peripheral blood samples (4 mL) were collected from each patient at the time of diagnosis and sent to the laboratory within 4 hours after collection. During the patient recruitment period, we also recruited 89 healthy volunteers as controls from the general population and collected their blood samples.

All 49 ENKTL patients received chemotherapy, 26 of whom were administered at West China Hospital. From these 26 patients we again sampled peripheral blood after two courses of chemotherapy. Treatment response was categorized as complete remission (CR), partial remission (PR), stable disease (SD), or disease progression (PD).^19^

### 2.2 Flow cytometry analysis

Blood aliquots (500 μL) were transferred to a 1.5‐ml Eppendorf tube, treated with ACK erythrocyte lysis solution(Leagene, Beijing, China), washed twice with phosphate‐buffered saline (PBS), and incubated in the dark at 4°C for 15 minutes with antibodies (eBioscience, CA, USA) against CD4 (conjugated to the fluorophore APC), CD8 (PE‐Cy7), PD‐1 (FITC), or PD‐L1 (PE). The samples were washed twice with PBS, resuspended in 300 μL PBS, and examined by flow cytometry (Navio Beckman Coulter, CA, USA). Data were analyzed using FlowJo X 10 software (Beckman Coulter).

### 2.3 Cell separation, RNA extraction, and mRNA reverse transcription

Blood aliquots (500 μL) were centrifuged at 3000 rpm to separate plasma from cellular components (lower layer). The upper plasma layer was transferred to a fresh tube and frozen at −80°C for subsequent sPD‐L1 assay (see below). PBMCs were separated from the lower cellular components by Ficoll density gradient centrifugation, mixed with 1 mL of Trizol reagent (MRC, Cincinnati, OH, USA) and stored at −80°C until assay. RNA was extracted from thawed samples using a Nanodrop spectrophotometer (Thermo Fisher Scientific, Waltham, USA) and 1 μg of total RNA was reverse‐transcribed into cDNA in a 20 μL reaction using of a reverse transcription kit (Takara, Dalian, China).

### 2.4 Quantitative RT‐PCR

Levels of mRNA encoding PD‐1 or PD‐L1 were measured by quantitative RT‐PCR using quantitative PCR kits (Takara, Dalian, China). The following primers were designed to target the PD‐1 or PD‐L1 transcript (NCBI Sequence Database). PD‐L1 forward, 5′‐CTATGGTGGTGCCGACTACA‐3′, PD‐L1 reverse, 5′‐TGCTTGTCCAGATGACTTCG‐3′, and PD‐1 forward, 5′‐GCGTGACTTCCACATGAGC‐3′, and PD‐1 reverse, 5′‐GCAGGCTCTCTTTGATCTGC‐3′. Reactions were cycled 39 times on a CFX Manager Detector (Bio‐Rad, CA, USA) with pre‐denaturation at 95°C for 15 minutes, denaturation at 94°C for 15 seconds, annealing at 55°C for 30 seconds, and extension at 70°C for 30 seconds. Relative levels of PD‐1 or PD‐L1 mRNA were calculated by the 2^−∆∆Ct^ method and normalized to levels of β‐actin mRNA. Each test was performed in triplicate.

### 2.5 ELISA of soluble PD‐L1 in plasma

Plasma fractions obtained as described above were thawed and assayed for sPD‐L1 using the Human PD‐L1 ELISA kits (catalog no. 28‐8, Abcam, Cambridge, UK), which has a manufacturer‐specified minimal detectable concentration of 2.91 pg/mL. First, samples were added to microplates pre‐coated with anti‐PD‐L1 monoclonal antibodies. After washing out the reagent and unbound antibody, TMB substrate was added to the wells, which were incubated for 10 minutes. Then stop solution was mixed to prevent blue color development, and the color changed to yellow. Absorbance at 450 nm was measured immediately using a microplate reader (R&D, MN, USA). Each test was performed in duplicate.

### 2.6 Statistical analyses

Data were analyzed using SPSS 24.0 (IBM, Chicago, IL, USA) and GraphPad Prism 6.0 software (GraphPad Software Inc., San Diego, CA, USA). Differences between groups in baseline clinical characteristics were assessed for significance using the Mann‐Whitney *U* test. Levels of PD‐1 and PD‐L1 on CD4+ or CD8+T cells, as well as levels of PD‐L1 mRNA and sPD‐L1 were expressed as median and interquartile range (IQR). Differences between groups were assessed for significance using the Kruskal‐Wallis test. For the subset of patients sampled at diagnosis and after two treatment courses, we assessed differences in levels of PD‐L1 mRNA or sPD‐L1 using the Wilcoxon matched‐pairs signed rank test. Univariate analysis to identify predictors of treatment response was performed using the Mann‐Whitney *U* test for continuous variables or chi‐squared test for categorical variables. Variables that were significant in univariate analyses were entered into a binary logistic regression multivariate model. Correlations between variables were analyzed using Spearman rank correlation. Differences associated with a two‐sided *P* < .05 were considered significant.

## 3 RESULTS

### 3.1 Baseline clinical characteristics of patients

Baseline clinical characteristics of patients with ENKTL or DLBCL are summarized in Table 1. In the ENKTL group, 12 (24%) were in stage III or IV, and 16 (33%) had lactate dehydrogenase (LDH) levels>250 U/L. All patients received at least two cycles of chemotherapy before radiotherapy. Fourteen (29%) patients received anthracycline‐containing chemotherapy regimens and the others accepted non‐anthracycline‐containing regimens. After two courses of chemotherapy, 21 patients (43%) achieved CR. We also recruited 89 healthy volunteers (46 men and 43 women) as the control group, whose median age was 41 years (IQR, 33.5‐53.5) (data not shown).

**TABLE 1:** Baseline and clinical characteristics of 123 patients

Characteristics	ENKTL patients (n = 49)	DLBCL patients (n = 74)
Age, year		
>60	8 (16)	30 (41)
≤60	41 (84)	44 (59)
Sex		
Male	32 (65)	34 (46)
Female	17 (35)	40 (54)
Ann Arbor Stage		
I‐II	37 (76)	49 (66)
III‐IV	12 (24)	25 (34)
Bulky mass		
Yes	5 (10)	19 (26)
No	44 (90)	55 (74)
B symptoms		
Yes	28 (57)	23 (31)
No	21 (43)	51 (69)
Extranodal sites		
>1	39 (80)	18 (24)
≤1	10 (20)	56 (76)
ECOG PS		
≥1	7 (14)	23 (31)
<1	42 (86)	51 (69)
Bone marrow involvement		
Yes	3 (6)	4 (5)
No	46 (94)	70 (95)
Lactate dehydrogenase (U/L)		
>250	16 (33)	29 (39)
≤250	33 (67)	45 (61)
EBER(+)		
Yes	49 (100)	3 (4)
No	0 (0)	71 (96)
PINK‐E and IPI		
Low (0–2)	34 (69)	56 (76)
High (≥3)	15 (31)	18 (24)
Chemotherapy		
Anthracycline	14 (29)	–
Non‐anthracyline	35 (71)	–
Response after two treatment courses		
Complete remission	21 (43)	–
Other	28 (57)	–

*Note*: Values are n (%).

Abbreviations: DLBCL, diffuse large B‐cell lymphoma; ECOG PS, Eastern Cooperative Oncology Group performance score; ENKTL, extranodal natural killer/T‐cell lymphoma; PINK‐E, prognostic index of extranodal natural killer/T‐cell lymphoma; IPI, international prognostic index; “–”, data not collected.

### 3.2 Expression of PD‐1 and PD‐L1 on CD4+ or CD8+T cells and relationships with clinical variables

**FIGURE 1:**
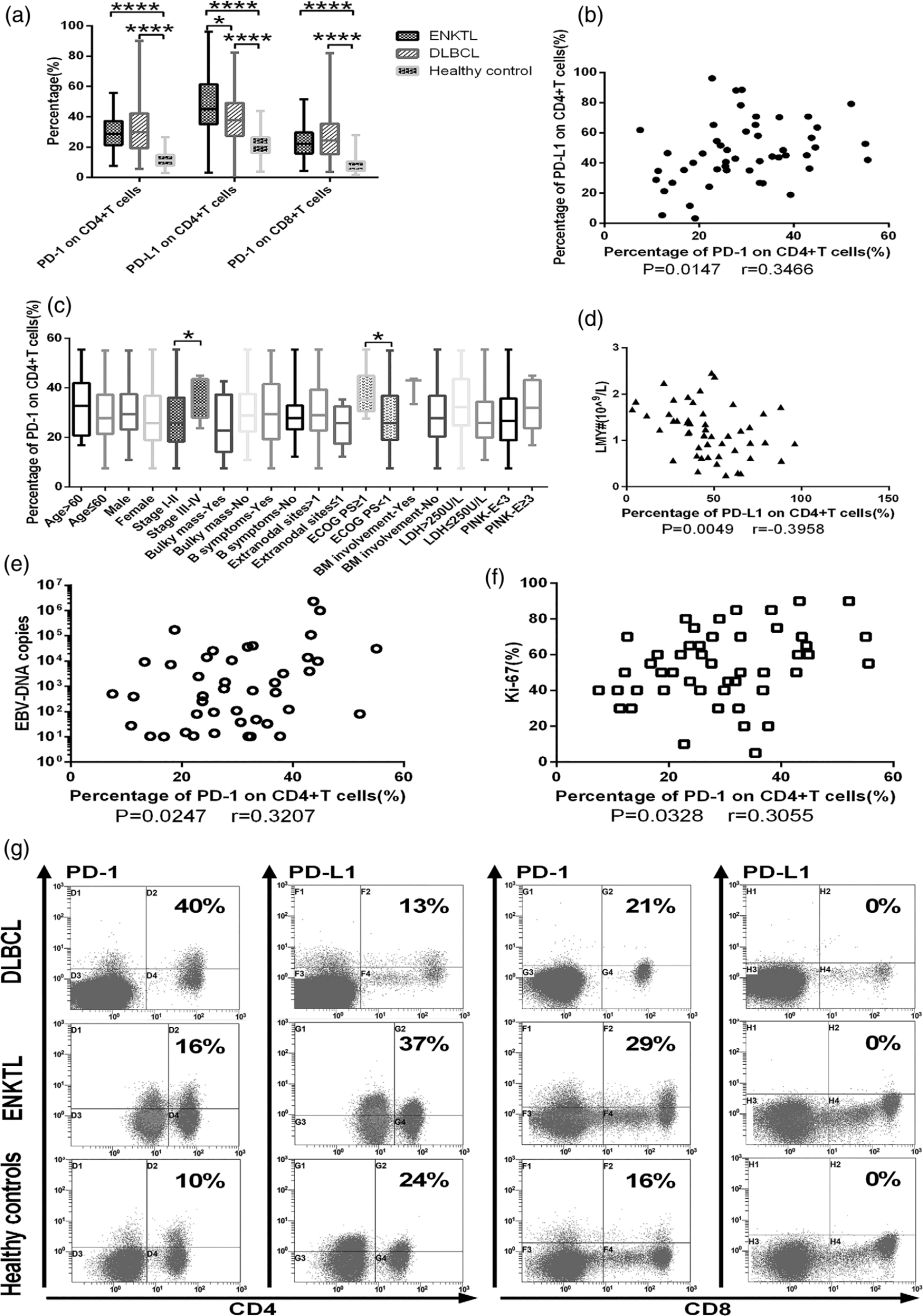
A, Positive percentages of CD4+ or CD8+ T cells expressing PD‐1 or PD‐L1 from extranodal natural killer/T‐cell lymphoma (ENKTL) or diffuse large B‐cell lymphoma (DLBCL) or from healthy volunteers. B, Correlation between PD‐1 and PD‐L1 expression on CD4+ T cells from ENKTL patients. C‐F, Correlation of PD‐1 level of CD4+ T cells in ENKTL patients with C, disease stage and C, Eastern Cooperative Oncology Group performance (ECOG) score, D, lymphocyte count, E, number of Epstein‐Barr virus genome copies, and F, Ki‐67 index. G, Representative flow cytometry plots showing expression of PD‐1 or PD‐L1 on CD4+ or CD8+ T cells from one DLBCL patient, one ENKTL patient and one healthy control. ****P < .0001, **P < .01, *P < .05

Flow cytometry (Figure 1G) showed that patients with ENKTL or DLBCL expressed higher levels of PD‐1 on CD4+ or CD8+T cells than healthy controls, as well as higher levels of PD‐L1 on CD4+T cells (Table 2, Figure 1A). Levels of PD‐L1 on CD4+ T cells were higher in ENKTL patients (Figure 1A). PD‐L1 was barely detectable on peripheral blood CD8+ T cells from either group of patients or from healthy controls (data not shown). There was a positive correlation between the expression of PD‐1 and PD‐L1 on CD4+T cells in ENKTL (Spearman *r* = 0.3466, *P* = .0147, Figure 1B). In addition, PD‐1 expression on CD4+ T cells differed significantly between patients in different disease stages (stages III‐IV vs I‐II, *P* = .0208, Figure 1C), with different Eastern Cooperative Oncology Group (ECOG) scores [>1 vs ≤1, 35.40 (30.60‐44.90) vs 25.85 (19.00‐36.83), *P* = .0161, Figure 1C], with different copy numbers of Epstein‐Barr virus (EBV) genome (Spearman *r* = 0.3207, *P* = .0247, Figure 1E), or with different Ki‐67 indices (Spearman *r* = 0.3055, *P* = .0328, Figure 1F). PD‐L1 expression on CD4+ T cells was associated with lymphocyte count (Spearman *r* = −0.3958, *P* = .0049, Figure 1D).

**TABLE 2:** Expression of PD‐1 and PD‐L1 on CD4+ or CD8+T cells, based on flow cytometry

Group	N	CD4+ cells positive for PD‐1, %	*P*	CD4+ cells positive for PD‐L1, %	*P*	CD8+ cells positive for PD‐1, %	*P*
ENKTL	49	28.80 (21.40‐37.30)	<.0001	45.00 (35.10‐61.40)	<.0001	22.10 (15.90‐29.65)	<.0001
DLBCL	74	29.90 (19.38‐42.23)	<.0001	37.80 (27.4549.10)	<.0001	24.30 (15.28‐35.43)	<.0001
Healthy controls	89	11.10 (7.80‐14.80)	–	21.50 (16.15‐26.50)	–	7.10 (4.45‐10.45)	–

*Note*: Values are median (interquartile range).

Abbreviations: DLBCL, diffuse large B‐cell lymphoma; ENKTL, extranodal natural killer/T‐cell lymphoma.

### 3.3 PD‐L1 mRNA and relationships with clinical variables

**FIGURE 2:**
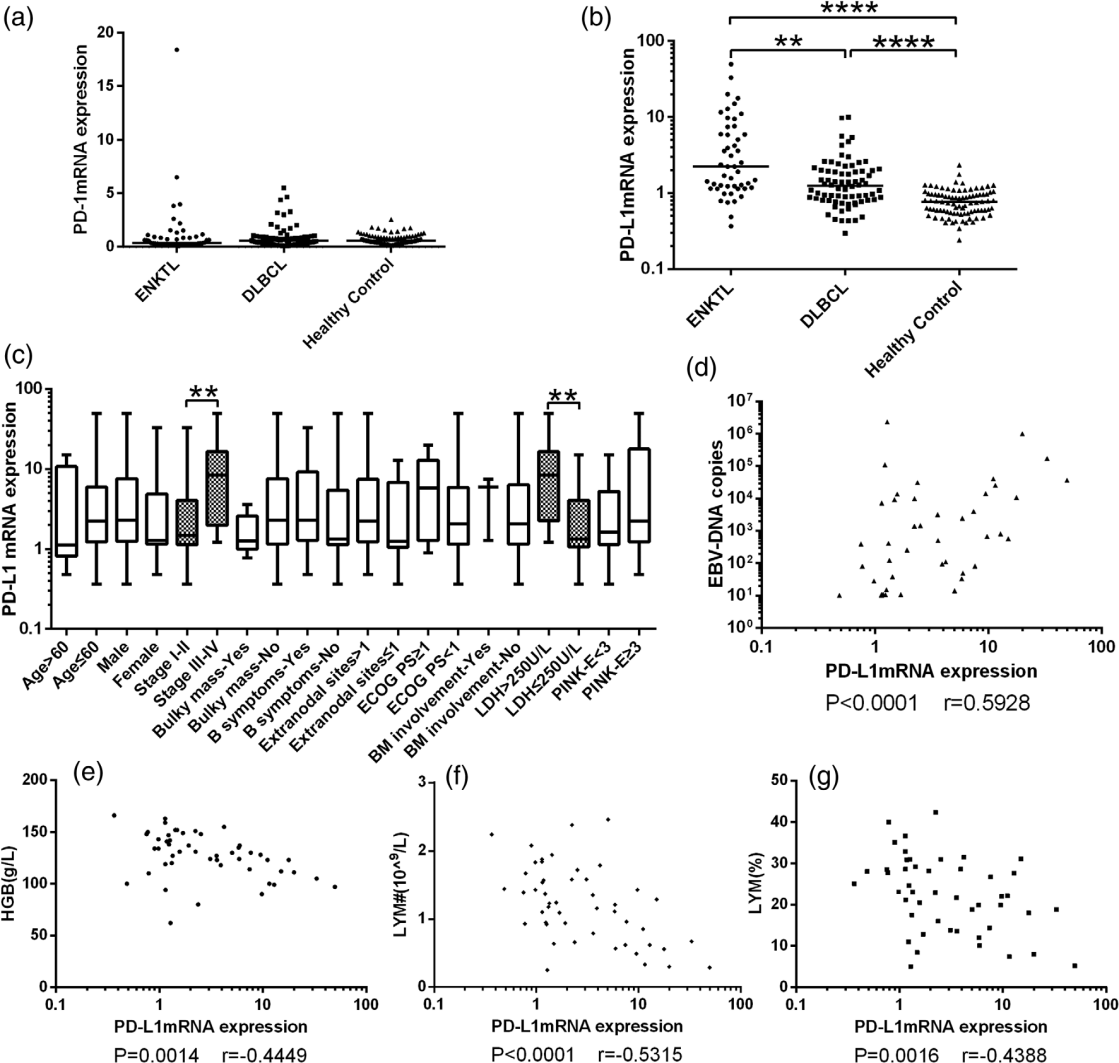
A, Levels of PD‐1 mRNA in peripheral blood mononuclear cells (PBMCs) were similar among patients with ENKTL or DLBCL and healthy controls. B, Levels of PD‐L1 mRNA in PBMCs were significantly higher in ENKTL patients [2.23(1.16‐6.70)] and DLBCL patients [1.25(0.81‐1.99)] than in healthy controls [0.77(0.56‐0.96)]. Levels were also higher in ENKTL patients than in DLBCL patients. C‐G, In ENKTL patients, levels of PD‐L1 mRNA in PBMCs varied with C, Ann Arbor stage and level of lactate dehydrogenase (LDH), D, copy number of Epstein‐Barr virus genome, E, hemoglobin, F, lymphocyte count, and G, lymphocyte percentage. ****P < .0001, **P < .01. DLBCL, diffuse large B‐cell lymphoma; ENKTL, extranodal natural killer/T‐cell lymphoma

Levels of PD‐L1 mRNA in PBMCs were significantly higher in ENKTL patients [2.23 (1.16‐6.70), *P* < .001] and DLBCL patients [1.25 (0.81‐1.99), *P* < .001] than in healthy controls [0.77 (0.56‐0.96)] (Figure 2A,B). Levels of PD‐L1 mRNA were significantly higher in ENKTL patients than in DLBCL patients (*P* < .001, Figure 2B). Among ENKTL patients, levels of PD‐L1 mRNA were significantly higher with more advanced stage [stage s III‐IV vs I‐II, 8.46 (1.99‐16.53) vs 1.48 (1.14‐4.06), *P* = .0032, Figure 2C], higher levels of lactate dehydrogenase (Figure 2C), greater number of EBV genome copies (Spearman *r* = 0.5928, *P* < .001, Figure 2D), lower hemoglobin level (Spearman *r* = −0.4449, *P* = .0014, Figure 2E), lower lymphocyte count (Spearman *r* = −0.5315, *P* < .001, Figure 2F), and lower lymphocyte percentage (Spearman *r* = −0.4388, *P* = .0016, Figure 2G).

### 3.4 sPD‐L1 and relationships with clinical variables

**FIGURE 3:**
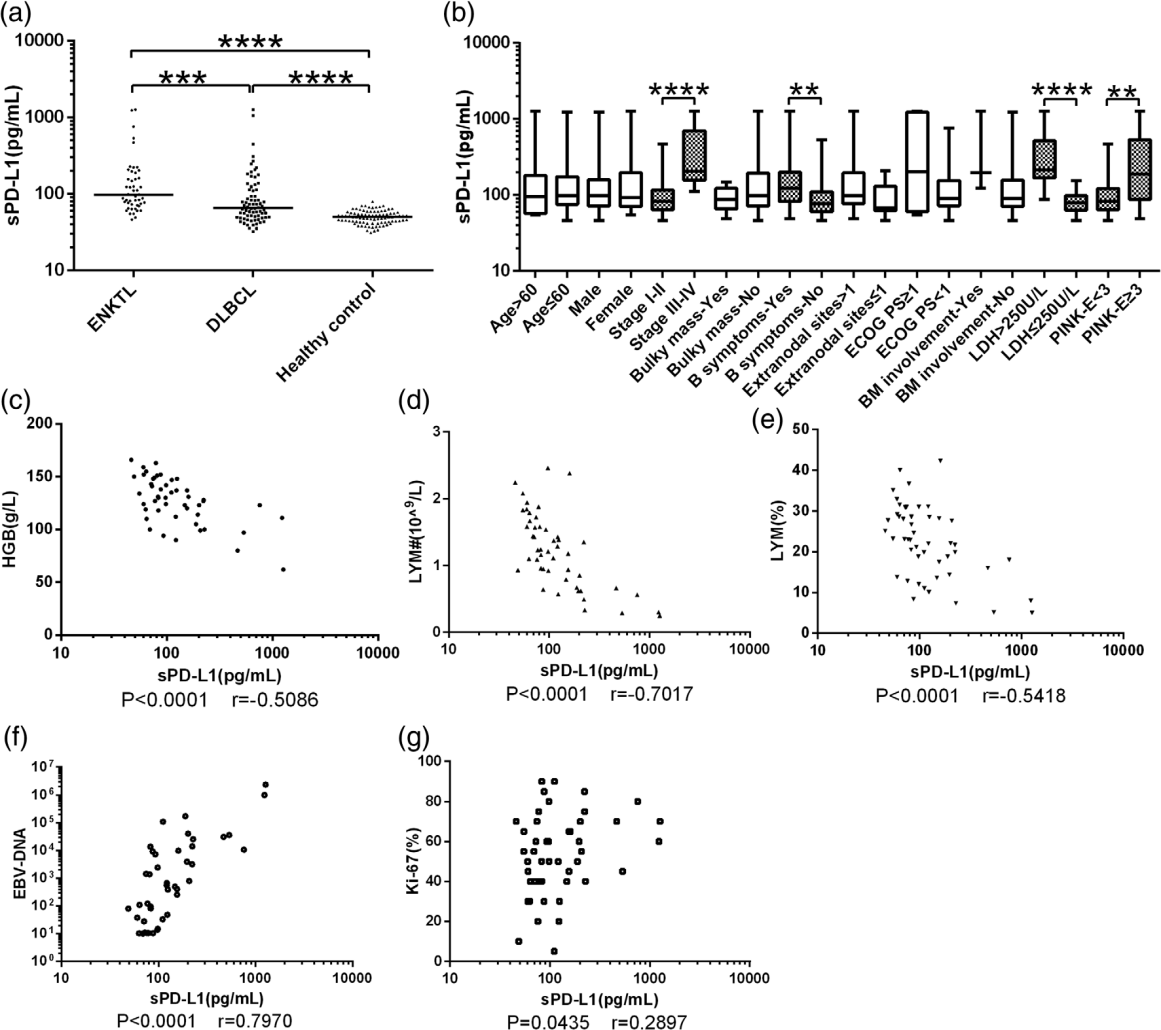
A, Levels of soluble PD‐L1 (sPD‐L1) were significantly higher in patients with ENKTL [97.29 (71.67‐174.32)] or DLBCL [65.12 (49.02‐113.23)] than in healthy controls [49.97 (42.93‐56.70)]. Levels were significantly higher in ENKTL patients than in DLBCL patients. B‐F, In ENKTL patients, sPD‐L1 level varied with B, Ann Arbor stage, B, B symptoms, B, lactate dehydrogenase (LDH) concentration, B, PINK‐E scores, C, hemoglobin, D, lymphocyte count, E, lymphocyte percentage, F, copy number of Epstein‐Barr virus genome, and G, Ki‐67 index. ****P < .0001, ***P < .001, **P < .01. DLBCL, diffuse large B‐cell lymphoma; ENKTL, extranodal natural killer/T‐cell lymphoma

**FIGURE 4:**
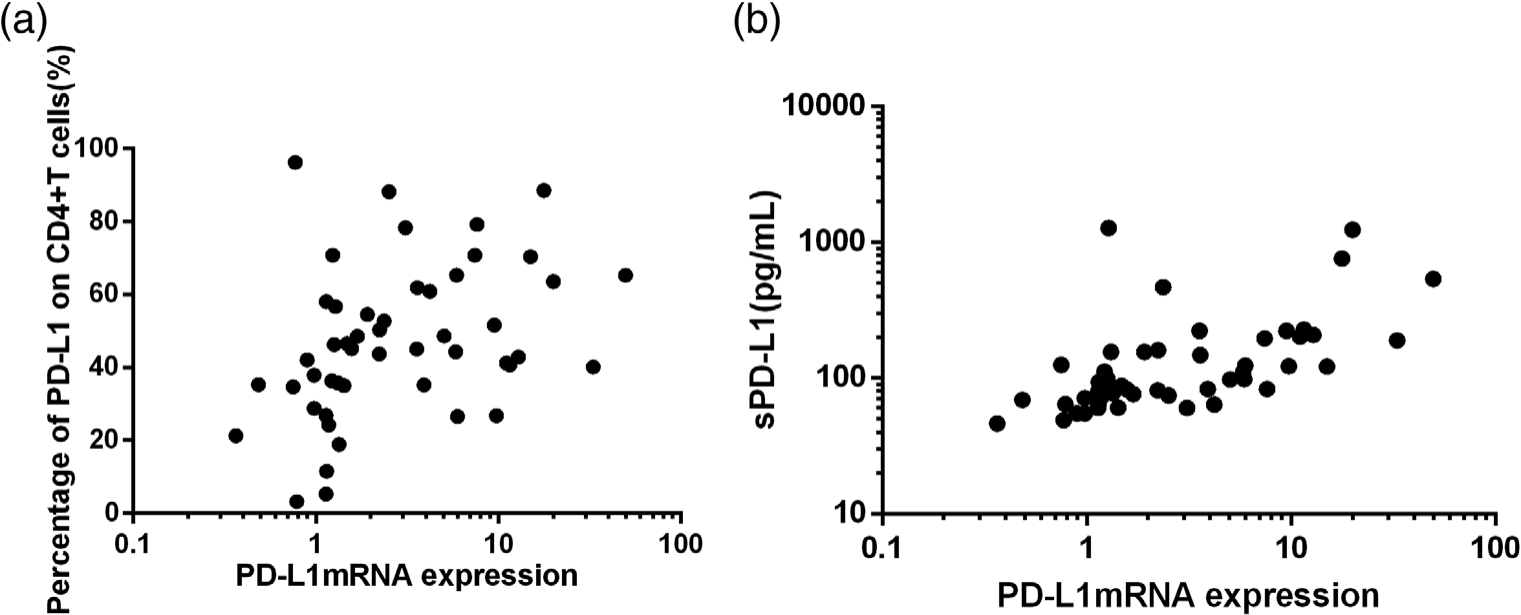
A, Correlation between PD‐L1 mRNA levels in PBMCs and PD‐L1 levels on CD4+T cells from ENKTL patients. B, Correlation between PD‐L1 mRNA levels in PBMCs and sPD‐L1 levels. PBMCs, peripheral blood mononuclear cells

Levels of sPD‐L1 (pg/mL) were significantly higher in patients with ENKTL [97.29 (71.67‐174.32), *P* < .0001] or DLBCL [65.12 (49.02‐113.23), *P* < .0001] than in healthy controls [49.97 (42.93‐56.70), Figure 3A]. Levels were significantly higher in ENKTL patients than in DLBCL patients (*P* < .001, Figure 3A). Among ENKTL patients, sPD‐L1 level was significantly higher in more advanced disease [stages III‐IV vs I‐II, 204.30 (156.4‐702.5) vs 82.52 (63.91‐115.90), *P* < .001, Figure 3B] or in the presence of B symptoms [yes vs no, 123.40 (82.65‐200.20) vs 77.27 (60.32‐110.70), *P* = 0.0062, Figure 3B], higher serum levels of lactate dehydrogenase [>250 vs ≤250 U/L, 214.1 (167.20‐517.60) vs 78.54 (63.34‐98.21), *P* < .001, Figure 4B], PINK‐E scores [≥ 3 vs < 2, 188.50 (87.59‐534.30) vs 83.04 (64.05‐122.30), *P* = .0041, Figure 3B], lower hemoglobin level (Spearman *r* = −0.5086, *P* < .001, Figure 3C), lower lymphocyte count (Spearman *r* = −0.7017, *P* < .001, Figure 3D), lower lymphocyte percentage (Spearman *r* = −0.5418, *P* < .001, Figure 3E), greater number of EBV genome copies (Spearman *r* = 0.7970, *P* < .001, Figure 3F), and higher Ki‐67 index (Spearman *r* = 0.2897, *P* = .0435, Figure 3G).

### 3.5 Correlation between PD‐1 or PD‐L1 biomarkers

Among the ENKTL patients, Spearman rank correlation analysis showed that PD‐L1 mRNA levels in PBMCs correlated with PD‐L1 levels on CD4+ T cells (*P* = .0021, Spearman *r* = 0.4298, Figure 4A) and with sPD‐L1 in plasma (*P* < .001, Spearman *r* = 0.6671, Figure 4B).

### 3.6 Association of PD‐1 or PD‐L1 biomarkers with treatment response

**FIGURE 5:**
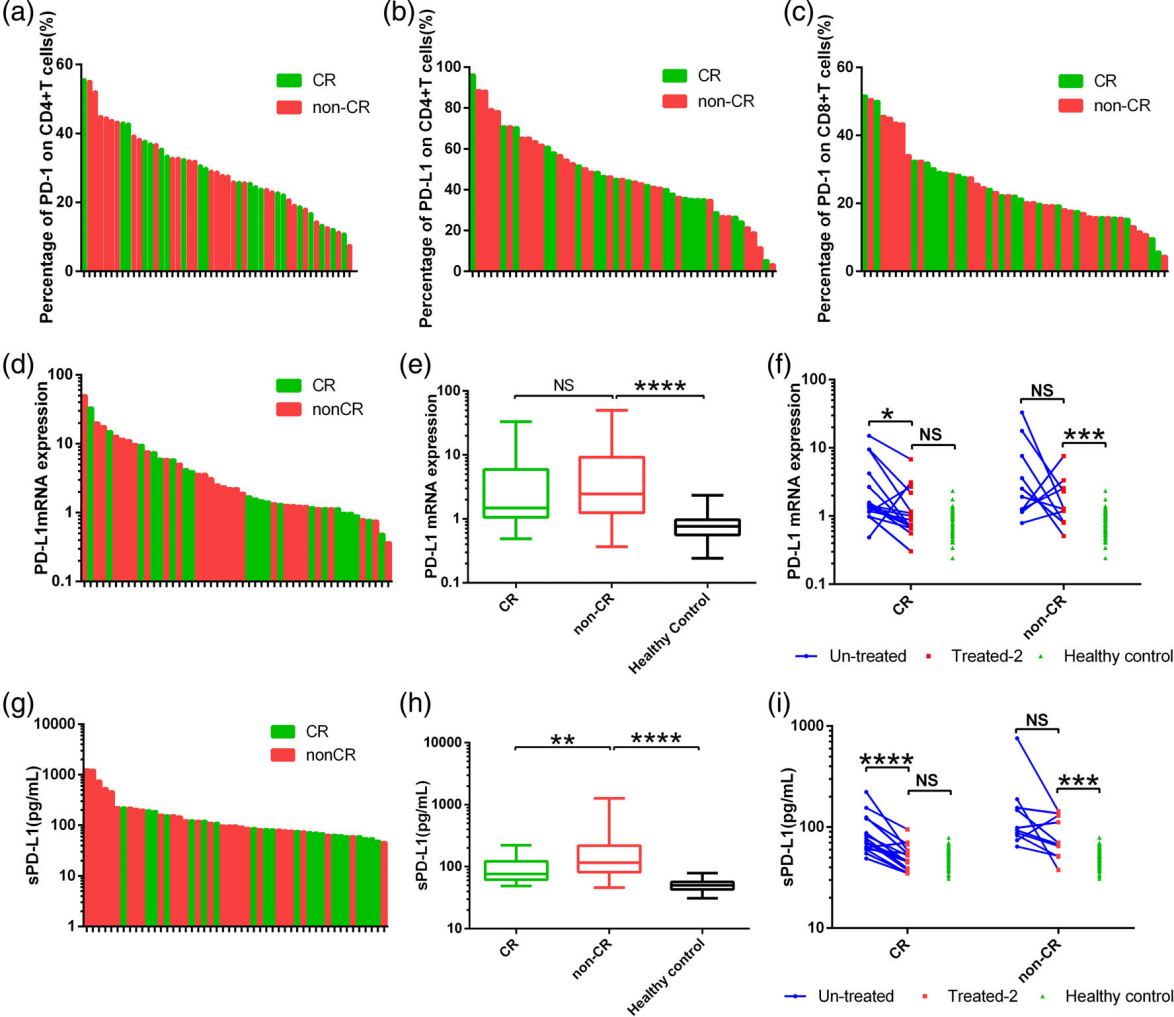
ENKTL patients who showed complete remission (CR) or a different response (non‐CR) after two rounds of treatment did not differ significantly in post‐treatment expression of A, PD‐1 on CD4+ T cells, B, PD‐L1 on CD4+ T cells, or C, PD‐L1 on CD8+ T cells in peripheral blood. D‐E, The CR and non‐CR response groups showed similar pretreatment levels of PD‐L1 mRNA. F, In the CR group, two rounds of treatment reduced the levels of PD‐L1 mRNA to the levels in controls. G‐H, The CR group showed statistically lower pretreatment levels of sPD‐L1 than the non‐CR group. I, In the CR group, two rounds of treatment reduced the levels of sPD‐L1 to the levels in controls. *P < .05, **P < .01, ***P < .001, ****P < .0001. ENKTL, extranodal natural killer/T‐cell lymphoma; NS, not significant

After two courses of treatment, 21 of the 49 ENKTL patients showed CR; 6, PR; 10, SD; and 12, PD. The rates of CR or other treatment response did not differ significantly among patients expressing different levels of PD‐1 or PD‐L1 on CD4+ or CD8+ T cells (Figure 5A‐C). Before treatment, the CR group showed a similar level of PD‐L1 mRNA in plasma as the other treatment response groups [1.48 (1.06‐5.90) vs 2.44 (1.24‐9.23), *P* = .2890] (Figure 5D,E), but that group showed a significantly lower sPD‐L1 level [75.95 (61.72‐122.3) vs 116.5 (81.48‐217.8), *P* = .0085] (Figure 5G,H).

Of the 26 ENKTL patients for whom we had blood samples after two courses of treatment, 16 showed CR; 5, PR; 3, SD; and 2, PD. In the CR subgroup, levels of PD‐L1 mRNA in PBMCs were significantly higher before treatment than after [1.38 (1.15‐3.823) vs 0.81 (0.67‐1.93), *P* = .0155]. A similar result was observed for sPD‐L1 [71.67 (61.07‐112.9) vs 46.04 (37.86‐57.64), *P* < .001]. The post‐treatment levels of both biomarkers were similar to those in healthy controls.

Among the patients showing PR, SD, or PD, treatment did not significantly reduce either the level of PD‐L1 mRNA in PBMCs [before vs after, 2.22(1.21‐10.16) vs 1.27(0.81‐2.74), *P* = .2754] or sPD‐L1 in plasma [before vs after, 95.68 (80.86‐163.50) vs 68.81 (51.60‐131.80), *P* = .0645]. The post‐treatment levels of both biomarkers were significantly higher than in healthy controls (Figure 5F,I).

Univariate analysis showed that ENKTL patients with high sPD‐L1 levels at diagnosis were more likely to achieve CR (*P* = .0085, Table 3), as were patients with high levels of lactate dehydrogenase (*P* = .0168) and Ki‐67 index (*P* = .0027). In multivariate analysis, however, only Ki‐67 index varied significantly with treatment response (*P* = .013).

**TABLE 3:** Univariate and multivariate analyses to identify predictors of treatment response among ENKTL patients

Variable	Univariate analysis		*P*	Multivariate analysis		*P*
	Complete remission (n = 28)	Other treatment response (n = 21)		OR	95% CI	
Age > 60(years)			.6800			
Yes	3 (10.7)	4 (19.0)				
No	25 (89.3)	17 (81.0)				
Sex			.6649			
Male	19 (67.9)	13 (61.9)				
Female	9 (32.1)	8 (38.1)				
Ann Arbor stage			.2701			
I‐II	19 (67.9)	18 (85.7)				
III‐IV	9 (32.1)	3 (14.3)				
Bulky mass			.7334			
Yes	3 (10.7)	2 (9.5)				
No	25 (89.3)	19 (90.5)				
B symptoms			.5597			
Yes	17 (60.7)	11 (52.4)				
No	11 (39.3)	10 (47.6)				
Extranodal sites			.3844			
>1	24 (85.7)	15 (71.4)				
≤1	4 (14.3)	6 (28.6)				
ECOG PS			.6800			
≥1	4 (14.3)	3 (14.3)				
<1	24 (85.7)	18 (85.7)				
Bone marrow involvement			.7964			
Yes	1 (3.6)	2 (9.5)				
No	27 (96.4)	19 (90.5)				
Lactate dehydrogenase>250 U/L			.0168	0.471	0.044‐5.056	.534
Yes	16 (57.1)	4 (19.0)				
No	12 (42.9)	17 (81.0)				
PINK‐E			.3709			
Low (0–2)	18 (64.3)	16 (76.2)				
High (≥3)	10 (35.7)	5 (23.8)				
Chemotherapy			.0552			
Anthracycline	5 (17.9)	9 (42.9)				
Non‐anthracyline	23 (82.1)	12 (57.1)				
Hemoglobin, g/L	125.5 (102.5‐141.0)	134.0 (118.5‐149.5)	.1454			
Platelet count, 10^9^ /L	202.5 (125.8‐296.8)	201.0 (169.5‐225.0)	.7755			
Lymphocyte count, 10^9^/L	1.22 (0.69‐1.58)	1.18 (0.80‐1.68)	.7223			
Lymphocyte percentage, %	22.05 (13.65‐28.05)	23.20 (13.65–28.05)	.3328			
Copies of Epstein‐Barr virus genome	1094 (31.43‐21 725)	48.20 (10.40‐2266)	.0548			
Ki‐67, %	62.50 (41.25‐73.75)	45.00 (30.00‐55.00)	.0027	0.951	0.914‐0.989	.013
CD4+ cells positive for PD‐1, %	28.90 (21.28‐39.05)	25.80 (20.40‐36.15)	.5378			
CD4+ cells positive for PD‐L1, %	47.45 (35.10‐64.85)	42.00 (35.10‐54.80)	.3856			
CD8+ cells positive for PD‐1, %	20.20 (15.85‐31.45)	22.30 (16.70‐29.65)	.7073			
PD‐L1 mRNA level	2.443 (1.241‐9.226)	1.481 (1.058‐5.896)	.2890			
sPD‐L1 level, pg/mL	116.5 (81.48–217.8)	75.95 (61.72–122.3)	.0085	0.990	0.973‐1.007	.242

*Note*: Values are n (%) or median (interquartile range).

Abbreviations: ECOG PS, Eastern Cooperative Oncology Group performance score; ENKTL, extranodal natural killer/T‐cell lymphoma; PINK‐E, Prognostic Index of extranodal natural killer/T‐cell lymphoma; OR, odd ratio; 95% CI, 95% confidence interval.

## 4 DISCUSSION

In this study, we found that levels of PD‐L1 mRNA and sPD‐L1 decreased to healthy controls after CR in ENKTL patients, suggesting that dynamic monitoring of PD‐L1mRNA and sPD‐L1 can be used to evaluate treatment response. In addition, patients in our sample who had higher sPD‐L1 levels before treatment were less likely to achieve CR, suggesting that sPD‐L1 levels can predict treatment response in patients with ENKTL.

Our results are consistent with a study showing that ENKTL patients had higher sPD‐L1 levels than healthy controls, and that higher levels correlated with worse prognosis.^16^ Our results are also consistent with work showing that ENKTL patients in stage I or II of the disease who had a high concentration of serum sPD‐L1 showed lower rate of CR to treatment and worse survival than those with a low sPD‐L1 concentration.^17^ The present work substantially extends those two studies because we measured biomarker levels before and after treatment in many of our patients, and we found higher sPD‐L1 levels in ENKTL patients than in DLBCL patients. This may help explain why ENKTL patients often show worse treatment response than DLBCL patients.^1^ We also identified lactate dehydrogenase level, Ki‐67 index, and sPD‐L1 level at diagnosis as predictors of treatment response in ENKTL patients, based on univariate analysis. Multivariate analysis, however, identified only Ki‐67 index as an independent predictor of treatment response. These results suggest that sPD‐L1 level can help predict treatment response in ENKTL, although it may not be a predictor on its own. By contrast, sPD‐L1 was found to be an independent predictor of metabolic response to immunochemotherapy in DLBCL patients.^20^ This discrepancy may reflect different sources and roles of sPD‐L1 in different subtypes of lymphoma.

Our study found that PD‐L1 was up‐regulated on CD4+ T cells in ENKTL patients. PD‐L1 can also be up‐regulated d on CD4+ regulatory T cells in Hodgkin lymphoma, which then inhibit the function of T cells expressing PD‐1.^21^ Therefore, we speculate that CD4+ T cells that strongly express PD‐L1 may bind to PD‐1 on other T cells to down‐regulate their response. Levels of PD‐1 on CD4+ or CD8+ T cells are increased in the peripheral blood of patients with gastric cancer^12^ or chronic lymphocytic leukemia.^15^ We found that PD‐1 and PD‐L1 expression on CD4+ T cells was elevated in ENKTL, which positively correlated with EBV DNA load, similar to a previous study.^22^ We further found a significant correlation between the expression of PD‐1 and PD‐L1 on CD4+T cells in ENKTL patients, and higher PD‐1 levels on CD4+ T cells correlated with more advanced high disease, higher ECOG score, higher copy number of the EBV genome, and higher Ki‐67 index. These results indicate that in ENKTL, CD4+ T cells expressing more PD‐1 are more important drivers of PD and tumor immune escape than T cells expressing less PD‐1. We found that PD‐L1 was barely expressed on circulating CD8+ T cells in patients with ENKTL or DLBCL and healthy volunteers. A previous study of patients with non‐small cell lung cancer also reported low rated of PD‐L1 expression ratio on CD8+ T cells (0.08%‐8.78%).^23^

We found higher PD‐L1 mRNA levels in ENKTL patients than in healthy controls, and the same has been reported in chronic lymphocytic leukemia^24^ and breast cancer.^25^ We further found that these higher PD‐L1 mRNA levels varied with disease stage, hemoglobin level, lymphocyte count, lactate dehydrogenase, and number of EBV genome copies. These results suggest that ENKTL patients expressing more PD‐L1 on circulating lymphocytes may have weaker systemic immunity and may therefore be more prone to PD.

Our study and several others suggest that sPD‐L1 level has prognostic potential. A multicenter clinical study demonstrated that patients with patients with higher plasma PD‐L1 level had worse prognosis than those with a lower level.^26^ Another study linked high sPD‐L1 level with more advanced gastric cancer staging and greater likelihood of lymph node metastasis.^27^ We also observed higher sPD‐L1 levels in patients with B symptoms, lymphopenia, more advanced disease, higher scores on the international prognostic index, higher Ki‐67 index, higher serum levels of lactate dehydrogenase, and lower hemoglobin concentration.

The EBV protein EBNA2 has been shown to up‐regulate expression of PD‐L1 in B‐cell lymphomas.^28^ Our study also found that the level of PD‐L1 mRNA in PBMCs and levels of sPD‐L1 in plasma correlated with EBV genome copy number of in peripheral blood. These findings further support the idea that the virus up‐regulates PD‐L1.

The source of up‐regulated sPD‐L1 in ENKTL is unclear. Myeloid suppressor cells have been proposed to be the main source of sPD‐L1 in this disease,^29^ but we found that sPD‐L1 level correlated positively with levels of PD‐L1 mRNA in PBMCs. This suggests that sPD‐L1 may be produced not only by tumor cells but also by certain non‐tumor immune cells.

Although small, our study proposes biomarkers that may facilitate prediction of treatment response in ENKTL. It also justifies further studies to elucidate how the PD‐1 signaling pathway is up‐regulated in ENKTL.

### 4.1 Ethics approval and consent to participate

This study was approved by the Ethics Committee of West China Hospital of Sichuan University (2018 review no.373). Participants provided written consent for their anonymized clinical data to be used and published for research purposes.

## CONFLICT OF INTEREST

The authors declare no conflicts of interest.

## Data Availability

The datasets generated and analyzed in the current study are available from the corresponding author on reasonable request.
